# Supplementary material for: An Augmented Reality Visor for Intraoperative Visualization, Guidance, and Temperature Monitoring Using Fluorescence
Source: J Biophotonics. 2024 Dec 23;18(2):e202400417. doi: 10.1002/jbio.202400417 (PMC11793943; doi:10.1002/jbio.202400417)
Supplement: Supplementary file 1 — Data S1. [file JBIO-18-e202400417-s001.pdf]

Supplementary Information

Augmented Reality Visor for IR- and NIR-Guided Laser Tissue Soldering Integrated in Surgical Workflows

Oscar Cipolato,[1-4] *Matthias Fauconneau,*[1] *Paige J. LeValley,*[1-4] *Robert Nißler,*[1-4] *Benjamin Suter,*[1-4] *Inge K. Herrmann*[1-4]*

[1] Nanoparticle Systems Engineering Laboratory, Institute of Energy and Process Engineering (IEPE), Department of Mechanical and Process Engineering (D-MAVT), ETH Zurich, Sonneggstrasse 3, 8092 Zurich, Switzerland.

[2] Particles Biology Interactions Laboratory, Department of Materials Meet Life, Swiss Federal Laboratories for Materials Science and Technology (Empa), Lerchenfeldstrasse 5, 9014 St. Gallen, Switzerland.

[3] The Ingenuity Lab, University Hospital Balgrist, Balgrist Campus, Forchstrasse 340, 8008 Zurich, Switzerland.

[4] University of Zurich, Faculty of Medicine, Rämistrasse 71, 8006 Zurich, Switzerland.

*ingeh@ethz.ch; inge.herrmann@empa.ch; +41 58 765 7153

Nanoparticle characterization

DLS measurement (Litesizer 500, Anton Paar) was used to measure the hydrodynamic size of the particles (Figure S1a).

Electron microscopy images confirm the findings from the DLS measures. SEM images were carried out using a scanning electron microscope (Magellan, Thermo Fisher Scientific) from ScopeM. The machine was operated using an acceleration voltage of 10 kV, beam current of 0.20 nA and a working distance of 10 mm. The following images show the structure of the ICG NPs after being dropcasted on a Si wafer (Figure S1b).

a

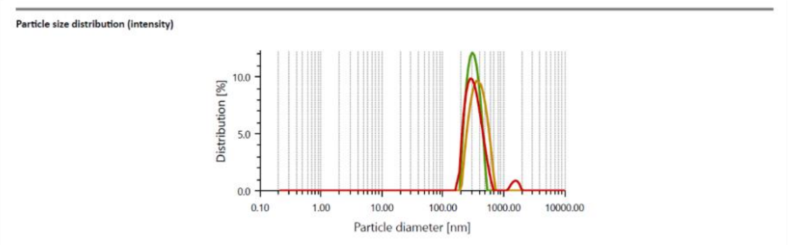

Measurements - Number

Index	Color	Hydrodyn. diam. [nm]	Polydisp. index [%]	Peak 1 [nm]	Peak 2 [nm]	Peak 3 [nm]	Transmittance [%]
1	green	355.2	21.7	225.5 (Number)	335.8 (Number)	- (Number)	73.2
2	orange	430.5	9.5	363.2 (Number)	- (Number)	- (Number)	74.2
3	red	387.3	9.3	297.4 (Number)	- (Number)	- (Number)	75.8

b

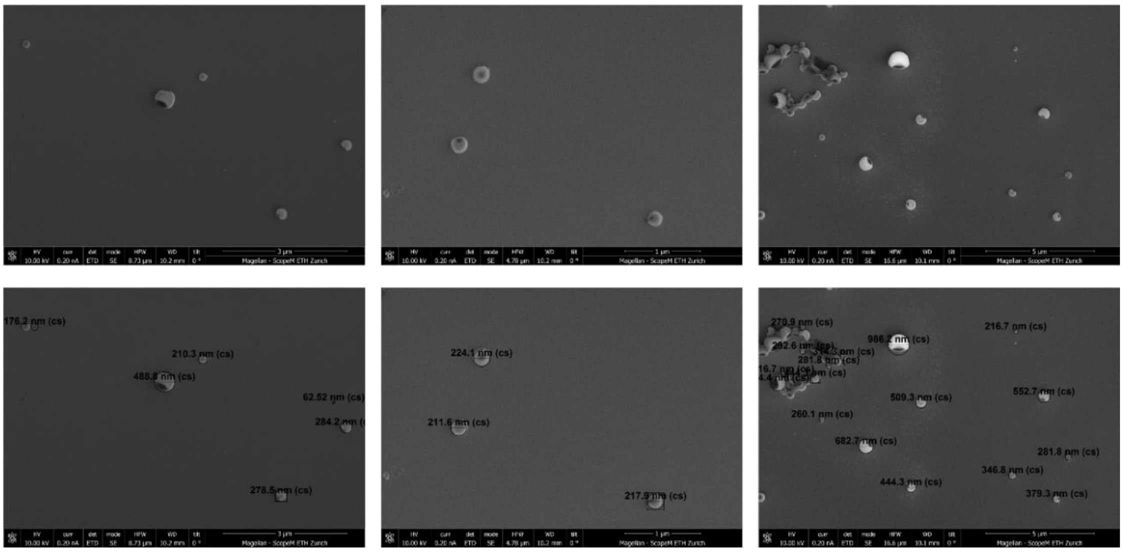

Weight and cost

An analysis of the weight of the headset allows to understand how much the setup will disturb the surgeon. Another important parameter is represented by the cost of the visor, which could hinder widespread adoption, especially in developing countries, if too high. The following table shows a breakdown of weight and cost for each component. (Table S1)

Table S1: breakdown of the approximate weight and cost of the various components of the headset.

**Item**	**Weight [g]**	**Cost [€]**
AR visor (excl. cables and controller)	70	700
3D printed add-on	20	Negligible
Laser safe lenses	10	200
NIR camera (x2)	20 (x2)	300 (x2)
Thermal camera	5	240
Optical filter: LP 700	2.5	130
Optical filter: BP 880	2.5	130
**Total headset**	**150**	**2000**
Laser setup (including accessories)	Not relevant	4000-5000
